# The profiles and tensile strength on straight roots of plants withstand transient tensile injured after self-repair

**DOI:** 10.1038/s41598-020-68358-8

**Published:** 2020-07-10

**Authors:** Chenglong Wang, Xin Zhang, Jing Liu, Bo Wang, Youfang Li, Qiang Li

**Affiliations:** 1 College of Desert Control Science and Engineering, Inner Mongolia Agricultural University, Hohhot, 010018 China; 2 Institute of Water Resources for Pastoral Area of the Ministry of Water Resources of China, Hohhot, 010020 China; 3 Environmental Management Office of Shendong Coal Group, Ordos, 017200 China

**Keywords:** Forestry, Plant ecology

## Abstract

Plants roots are severely injured during the process of withstanding transient tensile, and the injured roots can self-repair. We investigated the change law of the growth characteristics and tensile strength on straight roots withstand transient tensile injured after self-repair. The survival rate of two kinds of injured plants roots was between 60 and 89%. The test roots after self-repaired, the tensile strength reduction rate of *Hippophae rhamnoides* L. roots was greater than that of *Salix psammophila* roots. The tensile force was positively related to the power function of root diameter, the tensile strength was negatively related to the root diameter in a power function. The tensile strength of straight roots under small injured force showed an increasing trend, but the straight roots under the large injured force showed the opposite result. The survival rate of rough roots was greater than that of fine roots. The large injured force was not conducive to the repair and force again of the straight roots of two kinds of plants. The reduction rate of tensile strength after repaired with small force was less than that of large force. The self-repair ability of fine roots was weaker than that of rough roots.

Vegetation construction is an important measure to control soil erosion^1,2^. Compared with engineering measures, plant could be efficiently utilized in nature-based solutions to improve soil stability, vegetation measures have been recognised as an environmental-friendly and low CO_2_-emission solution for soil stabilisation^3,4^. Plants root and soil can form a kind of root–soil composites, which can fix and conserve soil, resist washing out and reduce erosion speed^5,6^. Plants are living and gradually growing, the effect of plant roots extensive distribution in the soil is like that of a steel bar in reinforced concrete. But the plant's roots have the self-reparability ability after injured force, so vegetation measures play an irreplaceable role for soil and water conservation. With the development of mechanical science and the requirement of vegetation construction, the mechanisms of soil reinforcement by plants roots have become the research hotspot^7,8^. Especially in coal mining subsidence area, there are many cracks in the surface, which leads to plants roots injure, and then influence plant growth and development^9^. Therefore, it is necessary and urgent to solve this issue in vegetation construction^10,11^.

Recently, research on the mechanism of soil erosion resistance of plant-roots mainly focuses on the material mechanical properties of single root^12–14^, the friction characteristics of root–soil interface^15,16^, and the shear characteristics of root–soil composite^17–21^. In particular, the research on the mechanical properties of single root is mainly based on the test method of material mechanics, to explore the ultimate tensile and tensile strength of straight root^22,23^ and the axial deformation elastoplastic characteristics^24^. Studies have shown that the axial tensile strength of single root varies by plant species^25^, the tensile strength of plant roots are important bio-mechanical traits that could be efficiently utilized to conserve soil and water^26^, the tensile force is proportional to the root diameter and the strength reverse^27^, the axial deformation of most plant roots show elastoplastic characteristics. The above research results can explain to some extent the mechanisms of soil reinforcement by straight roots, but the root system is used as an engineering material similar to steel bars in the test, to study the ultimate material mechanical properties of roots under transient tensile or short-term external forces. Plants roots have the self-repairability ability, but research only about the function of mycorrhizal fungi promote the repair of roots^28,29^ and the effects of coal mining on the roots growth of different specifications *Artemisia sphaerocephala* Krasch and its self-repairing ability^30^. The changes in mechanical characteristics of the injured roots after self-repair have not been reported, and related research on soil reinforcement has not yet been carried out.

The test plants, *Salix psammophila* and *Hippophae rhamnoides* L., are deciduous woody shrub species. The stems are woody with long branches, and are sand-resistant and buried. The main root are thick and long, with many lateral roots. They are also the main plant species to provide soil and water conservation in arid and semi-arid areas of northwest China. Therefore, it is very meaningful to study on the roots withstand force injured after self-repair. The context provide a reference for research methods on the study of plant roots for continuous soil reinforcement and provide the scientific basis for screening excellent plant species for local vegetation construction. The aim of this study is to validate that the plants roots have self-healing ability, the injury force conditions affect self-healing ability of roots.

## Materials and methods

### Study site

This study was conducted in Shenmu County of Shaanxi Province in China (110° 05′–110° 30′ E, 39° 27′–39° 15′ N), which is located in the continental arid and Semiarid areas. The annual average temperature is 8.9 °C. The annual frost-free period is 130 days, the mean annual precipitation of the area is about 396 mm, and potential evaporation is 1,790 mm.

The research plot is in the heartland of Shendong coal mining subsidence area in Shenmu County, typical steppe landscape, soil impoverishment, and fragile ecological environment. The basic physical soil properties in the test site were measured (Table 1). According to the SL237-1999 engineering classification standard of the Geotechnical Test Regulations, the soil in the test area was named as low liquid limit silt (ML). Major plant species under natural conditions in the study area include *S. psammophila*, *Caragana microphylla* Lam., *H. rhamnoides* L., *Artemisia ordosica* Krasch., *Agriophyllum squarrosum* (Linn. ) Moq., and *Lespedeza bicolor* Turcz.

**Table 1:** Basic physical properties of soil in the test area.

Moisture content (%)	Density (g cm^−3^)	Dry density (g cm^-3^)	Liquid limit (%)	Plastic limit (%)	Plasticity index (%)	Soil type
8.87	1.67	1.53	24.47	19.69	4.78	Silt

### Root sampling

The straight roots of 4 years old of *S. psammophila* and *H. rhamnoides* L. were used as materials, and applied instantaneous axial small injured force (corresponding to 30% of the average ultimate force of the radial level, less than the elastic ultimate force, and the deformation is recoverable elastic deformation) and instantaneous axial large injured force (corresponding to 70% of the average ultimate force of the radial level, greater than the elastic ultimate force, and the deformation is irreversible plastic deformation) without leaving the plant body, to understand the survival rate, the change in root diameter and tensile strength of straight roots withstand transient tensile injured after self-repair.

As the layers of soil could interfere with the anti-tension force and anti-tension strength of roots, we excavated the roots without leaving the plant body and selected the roots which are distributed in the same soil layer. Straight roots with uniform diameter ranged from 1 to 4.5 mm, roots segments of 100 mm were selected from the root systems. To sufficiently attribute the tensile ability of roots, the selected roots were divided into seven diameter classes with 0.5 mm interval. To ensure the parallelism of the test, each diameter class selected eight test roots and eight control roots.

**Figure 1:**
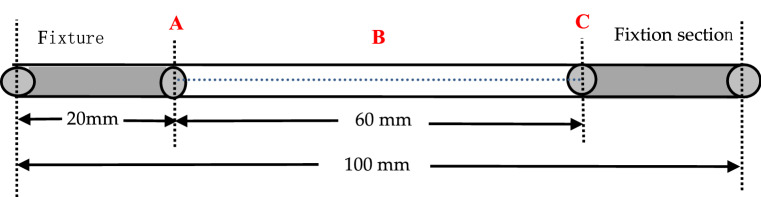
The schematic diagram of test root.

In the test, the soil around test roots was removed and the position of test roots was kept unchanged so that the exposed length of the roots reached the test requirements, the test roots were shaded, and sprayed water to maintain moisture. And each test root length was greater than 100 mm (Fig. 1), three points of A, B and C was selected along the root, and the diameters were measured by the cross method using an electronic Vernier caliper with an accuracy of 0.01 mm. B was the midpoint of the test root, A, C were the ends of the 30 mm from the midpoint. The diameters of control roots were measured in the same way.

### Root treatments

**Figure 2:**
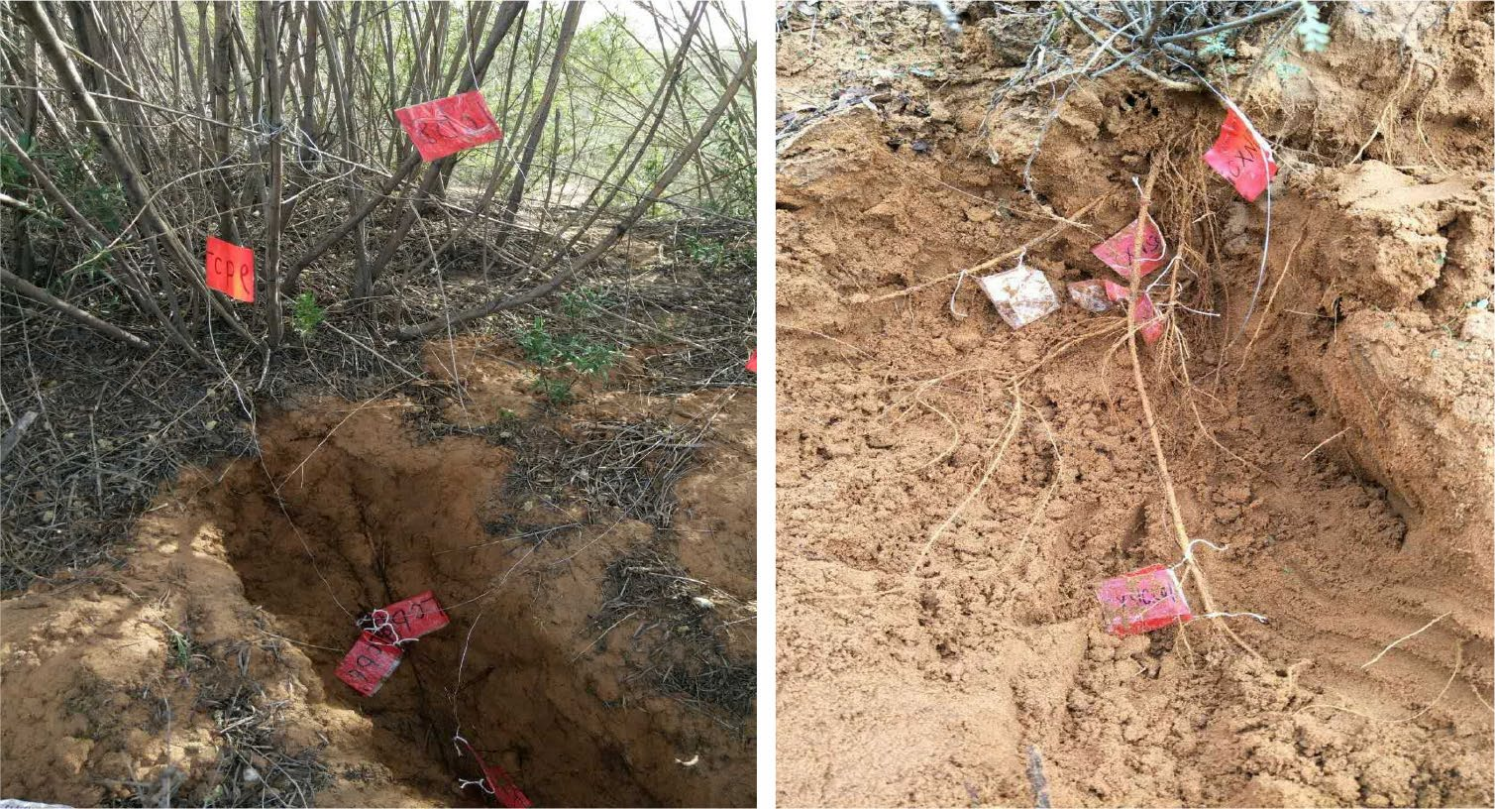
Excavation phase of test roots.

At the beginning of the plant growing season, in 2019 May. Test roots were applied two instantaneous axial injured forces without leaving the plant body, then covered soil growth. The process of excavation and covering soil was also carried out on the control roots in the same way (Fig. 1). By August, after a 3-month growth period, the test roots self-repaired for 3 months and excavated the test roots and control roots again, observed the survival rate, measured the root diameter and the tensile strength of test root (Fig. 2).

**Figure 3:**
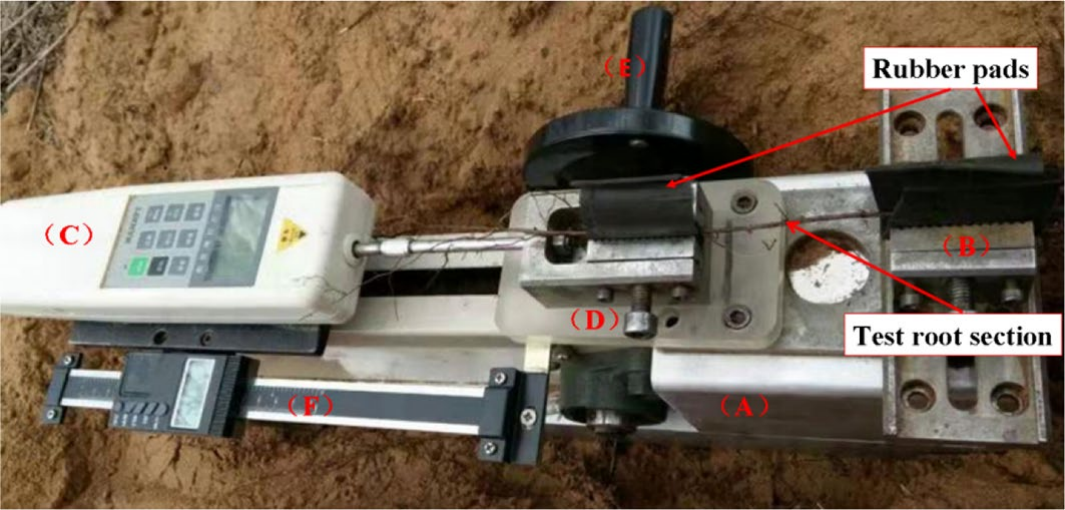
HG 100 digital display type push–pull meter and self-made portable test instrument.

Root tensile tests were conducted by a homemade portable instrument (Fig. 3). The instrument is composed with a platform (Part A), a root clap (Part B) fixed on the platform, a HG 100 digital display type push–pull meter (Part C), a moveable root clap (Part D) which is connected with Part C, a crank handle (Part E) which is used to move Part C and a Vernier caliper (Part F) which is connected with Part C to control the loading rate of the load. The test root is clapped by the two root pads. To make sure the test root not slip, we put a rubber pad inside each of the root clap. When the crank handle is turned, Part C is moved away from Part B and the force acted on the test root is recorded. The accuracy of HG 100 digital display type push–pull meter is 0.05 N. After selecting the test root, carefully excavated the soil under the test root and placed the instrument (length 50 cm, width 13 cm, height 20 cm). Fixed the points a and c of the test root at the jaws of the clamp so that the test root was in tension. The axial direction was pulled, and the length of the instrument was placed in the same direction as the root growth direction. Using 50 mm/min loading rate applied force injury by reading the Vernier caliper moving rate, stopped after the degree of injury urging force of the design, marked the ends of the test root segment and backfilled them, and marked on the ground to be dug again. The treatment method of the parallel control test roots was the same.

### Determination of injury force

The recoverable elastic deformation occurs in the root system before the elastic limit point. After the elastic limit point, the root system undergoes irreversible plastic deformation. Previous researches indicate that the elastic limit of the 0–8 mm straight root of *S. psammophila* is about 40% of the ultimate tensile strength, and that of *H. rhamnoides* L. is about 60% of the ultimate tensile strength^30,31^. Tests have shown that when the injury force reaches 80% of the average ultimate tensile strength, more test roots break when applying the injury forces. To observe impact of varied injury force on the self-repair of roots, we selected two levels of injury force in this study, the small injury force was 30% of the ultimate tensile force (less than the elastic limit point), and the large injury force was 70% of the ultimate tensile force (greater than the elastic limit point).

Due to the uneven root diameter along the axial direction, it was impossible to determine the fracture point at which the test root may be damaged before the test. To guarantee data quality, the measured number was not recorded when the test root was fractured in the experiment. The ultimate force was calculated from the regression equation according to the average root diameter of each test root test segment, the average root diameter of each test root was the mean of the root diameters of the three points A, B, and C. According to the root diameter of each test root, the ultimate tensile force was calculated by the regression equation, and the corresponding small injury force and large injury force were determined (Table 2).

**Table 2:** Ultimate anti-fracture force and its regression equation with root diameter of two plants.

Plant species	Root diameter (mm)	Ultimate tensile strength (N)	Regression equation
*Salix psammophila*	1.35–4.17	28.79–149.00	Y = 18.51x^1.495^, R^2^ = 0.992
*Hippophae rhamnoides*	1.23–4.27	12.26–99.00	Y = 10.14x^1.548^, R^2^ = 0.970

### Data analysis

The data were analyzed using SPSS 15.0 for Windows. The test roots and the parallel control roots were excavated after self-repaired for 3 months, observed the root shape, color and elasticity. If the root turned black, dry and begins to fall off, the root was dead. For the roots that survived, the root diameter and ultimate tensile strength were measured again, and the tensile strength was calculated using Eq. 1.

1 $$ {\text{P}} = 4{\text{F}}/\left( {\uppi {\text{D}}^{2} } \right) $$

where P is the tensile strength (MPa), F is the tensile force (N), D is the root diameter (mm).

## Results

### The survival rate and root diameter change after self-repair

The survival rate and root diameter change of straight roots after self-repair are shown in Table 3. The survival rate of straight roots withstands transient tensile injured after self-repair was 60–89%, and that of parallel control roots about 90%. Compared with parallel control, the survival rate of the two plants roots of 1.0–2.5 mm straight roots under the small injury force and the large injury force was decreased as follows: *S. psammophila* (8.36%, 25.00%), *H. rhamnoides* L. (12.47%, 29.95%). And that of 2.5–4.5 mm straight root under the small injury force and the large injury force was decreased as follows: *S. psammophila* (5.56%, 14.24%), *H. rhamnoides* L. (15.79%, 21.40%). The survival rates of test roots in the range of 1.0–4.5 mm diameter range after self-repair, the sequence is parallel control > small injury force > large injury force. To a certain extent, after the roots of the two plants were injured by the axial force, the ability of the self-repairing growth of the fine roots was greater than that of the coarse roots, and the effect of the large injury force on the growth of the straight roots of the two plants was more significant.

**Table 3:** Survival rate and root diameter growth rate of 2 plants after self-repair.

Plant species	Injury force	Root diameter (mm)	Number of test roots	Number of live roots after self-repair	Survival rate (%)	Root diameter growth rate %
*Salix psammophila*	Small injury force	1.0–2.5	25	21	84.00	19.29 ± 2.68
		2.5–4.5	19	17	89.47	13.35 ± 2.84
	Large injury force	1.0–2.5	32	22	68.75	17.98 ± 2.18
		2.5–4.5	16	13	81.25	11.17 ± 0.76
	Parallel control	1.0–2.5	24	22	91.67	25.03 ± 4.57
		2.5–4.5	19	18	94.74	15.43 ± 1.97
*Hippophae rhamnoides*	Small injury force	1.0–2.5	29	22	75.86	22.23 ± 3.04
		2.5–4.5	19	15	78.95	13.94 ± 0.93
	Large injury force	1.0–2.5	28	17	60.71	21.87 ± 3.10
		2.5–4.5	19	14	73.68	13.29 ± 0.92
	Parallel control	1.0–2.5	29	26	86.67	24.99 ± 3.60
		2.5–4.5	17	15	93.75	17.86 ± 1.96

### The differences in tensile ability of two plants roots after self-repair and that of before injured

**Figure 4:**
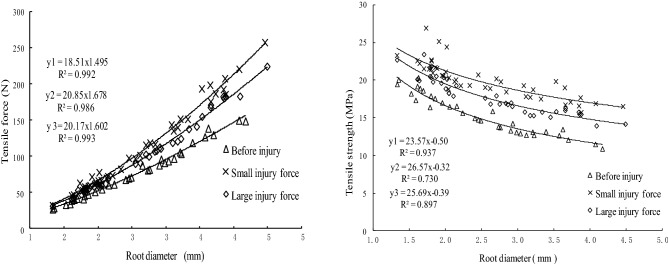
The differences in tensile ability of *Salix psammophila* roots after self-repair and that of before injured.

**Figure 5:**
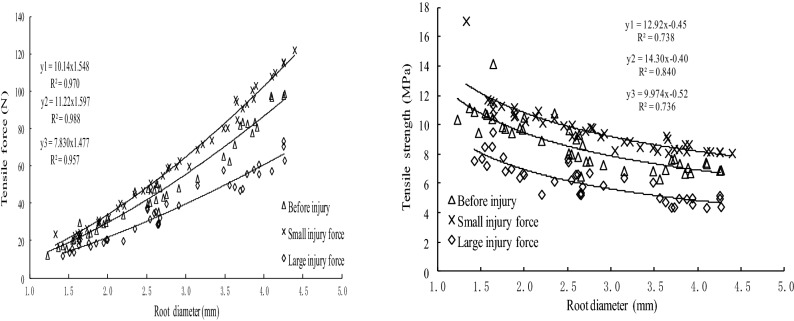
The differences in tensile ability of *Hippophae rhamnoides* L. roots after self-repair and that of before injured.

From Figs. 4 and 5, we can know that whether it is before injury or subjected to different injury forces after self-repair, the tensile strength of the straight root was positively related to the power function of the diameter. Although the tensile strength of each plant root was different before the injury and subjected to different injury forces after self-repair, the three tensile strength-root diameter curves were similar. That is, the intensity varied with the root diameter, and the tensile strength was negatively related to the root diameter in a power function. The finer the root diameter, the greater the tensile strength of straight root.

The difference on the ultimate tensile strength of straight roots between before injury and subjected to different injury forces after self-repair were related to the plant species and the degree of injury. The test roots of two plants were applied to the small injury force, the self-repairing process had better growth posture, and the tensile force and tensile strength were greater than that of before injury. But the test roots of two plants were applied to the large injury force, the self-repairing process, and the tensile strength after self-repaired varied by plant species. The tensile strength of the *S. psammophila* roots, which were applied the large injury force and self-repaired, was higher than that of before injured. But that of *H. rhamnoides* L. roots reversed, the tensile strength and tensile force of before injured was higher than that of injured and self-repair.

The root diameters of the two roots in the 1–4.5 mm diameter range were randomly distributed. It was impossible to guarantee the root diameters of the roots before and after the injured. The growth rate of the tensile strength of the roots of different diameter grades was calculated, to study the effect of the diameter on the tensile strength after self-repair. The results are shown in Table 4.

**Table 4:** The differences in tensile strength between 2 plants roots after self-repair and that of before injured.

Plant species	Root diameter (mm)	Tensile strength of the control roots before injury (MPa)	Tensile strength and growth rate after self-repair under different injury force			
			Small injury force (MPa)	Growth rate(%)	Large injury force (MPa)	Growth rate(%)
*Salix psammophila*	1.0–2.0	18.44 ± 1.22a	22.39 ± 1.72c	21.42	20.94 ± 1.23b	13.56
	2.0–3.0	14.77 ± 1.19a	20.05 ± 1.48c	35.75	17.83 ± 1.08b	20.72
	3.0–4.5	12.54 ± 0.81a	17.68 ± 1.19c	40.99	15.42 ± 0.67b	22.97
*Hippophae rhamnoides*	1.0–2.0	10.52 ± 1.24b	11.66 ± 1.85c	10.84	7.63 ± 0.93a	–27.47
	2.0–3.0	8.56 ± 1.25b	9.91 ± 0.54c	15.77	6.22 ± 0.79a	– 27.34
	3.0–4.5	7.07 ± 0.39b	8.45 ± 0.35c	19.52	4.97 ± 0.61a	– 29.70

The Duncan test (*P* < 0.05) showed that the tensile strength of the control roots before the injury of the two plants was significantly different from the tensile strength of the two roots after 3 months of self-repair. Compared with the tensile strength of the control roots before the injury, the tensile strength growth rate related to injury force, root diameter and plant species. The tensile strength growth rate of test roots under the small injury force after 3 months of self-repair was greater than that of under the large small injury force.

The different test roots were subjected to two levels of injury force, then self-repaired and measured the tensile strength, the tensile strength growth rate showed a positive correlation with the root diameter. The self-repairing effect of fine roots was weaker than that of coarse roots. And the thicker the test roots, the greater the growth rate of tensile strength. Within each diameter range, the growth rate of tensile strength of *H. rhamnoides* L roots after injuried by two levels of injury force were less than that of the *S. psammophila* roots. The ability of the *S. psammophila* roots to self-repair was stronger than that of the *H. rhamnoides* L roots.

### The differences in tensile ability of test toots and control roots

**Figure 6:**
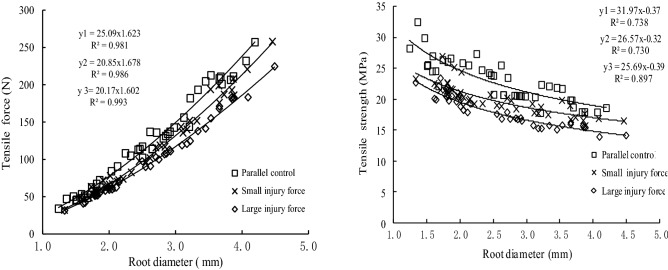
The differences in tensile ability between *Salix psammophila* roots after self-repair and parallel control roots.

**Figure 7:**
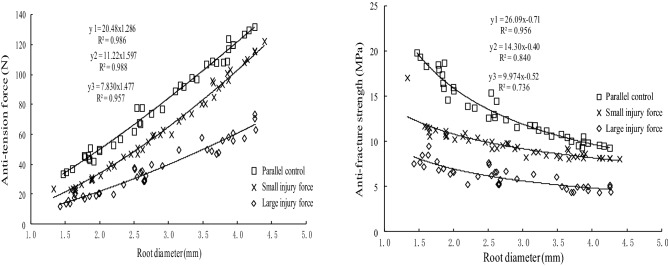
The differences in tensile ability between *Hippophae rhamnoides* L. roots after self-repair and parallel control roots.

Figures 6 and 7 show the differences in tensile ability between roots after self-repair and parallel control roots of the two studied species. From Figs. 5 and 6, the tensile force of control roots and test roots after self-repaired was positive to the power function of the root diameter and the tensile strength of the two types of roots was negative to the power function of the diameter.

The Duncan test (*P* < 0.05) showed that the tensile strength of the test roots after the self-repair of the two plants was significantly different from that of the parallel control roots. The tensile force and tensile strength of the test roots of the two plants were less than those of the parallel control roots under two levels of injury force, and that of subject to the large injury force was less than that of subject to the small injury force. The difference in tensile force, a tensile strength between the *H. rhamnoides* L. roots after self-repair and parallel control roots were greater than that of *S. psammophila* roots.

Compared with the tensile strength of the control roots (Table 5), the tensile strength reduction rate related to injury force, root diameter and plant species. In the same diameter range, the reduction rate of the tensile strength of the two plant test roots after repairing with small injury force was less than that of large injury force. Under the same injury force, the reduction rate of the tensile strength of the roots of the two plants decreased with the increase of the root diameter. The self-repairing effect of the coarse roots was stronger than that of the fine roots. Within each diameter range, the reduction rate of the tensile strength of *H. rhamnoides* L. roots after injury by two degrees of injury force was greater than that of corresponding sand willows, and the self-repairing ability of *H. rhamnoides* L. roots was weaker than that of *S. psammophila* roots.

**Table 5:** The differences in tensile strength between 2 plants roots after self-repair and the parallel control roots.

Plant species	Root diameter (mm)	Tensile strength of the control roots (MPa)	Tensile strength and induction rate after self-repair under different injury force			
			Small injury force (MPa)	Reduction rate (%)	Large injury force (MPa)	Reduction rate (%)
*Salix sammophila*	1.0–2.0	27.59 ± 2.69b	22.39 ± 1.72a	18.85	20.94 ± 1.23a	24.10
	2.0–3.0	22.92 ± 2.39c	20.05 ± 1.48b	12.52	17.83 ± 1.08a	22.21
	3.0–4.5	19.79 ± 1.76c	17.68 ± 1.19b	10.66	15.42 ± 0.67a	22.08
*Hippophae rhamnoides*	1.0–2.0	17.59 ± 0.63c	11.66 ± 1.85b	33.71	7.63 ± 0.93a	56.62
	2.0–3.0	13.57 ± 1.33c	9.91 ± 0.54b	26.97	6.22 ± 0.79a	54.16
	3.0–4.5	10.34 ± 0.82c	8.45 ± 0.35b	18.28	4.97 ± 0.61a	51.93

## Discussion

The tensile force of the roots of *S. psammophila* and *H. rhamnoides* L. in normal growth state is positively correlated with the root diameter, and the tensile strength is negatively correlated with the root diameter as a power function^32,33^. This is the same as the roots of *Pinus tabulaeformis* Carr., *Larix principis-rupprechtii* Mayr., *Betula platyphylla* Suk., *Quercus mongolicus* Fisch. ex Ledeb. and *Ulmus pumila* Linn^34,35^. After the root system is injured and self-repaired, the power function relationship between tensile force, tensile strength, and root diameter has not been changed.

The plant roots were supplied with instantaneous injured force, then after self-repaired for 3 months, the sequence of the root diameter growth rate and tensile strength is parallel control > small injury force > large injury force. This indicates that the axial injury force greater than the average elastic limit of the diameter grade is more harmful to the straight roots of the two plants than the injury force within the elastic limit range. This explains to a certain extent that the growth rate of the root diameter and the growth rate of the roots are less than the test roots of the small injury force in the elastic deformation stage after 3 months of repairing the large injury of the plant roots. That is, the large injury force that is subjected to plastic deformation causes the root fiber to be greatly injured, and thus has a great influence on the root growth.

After 3 months of self-repair, the roots of *S. psammophila* and *H. rhamnoides* L. can continue to survive under different levels of injury force. The survival rate and root diameter growth rate are as follows: parallel control > small injury force > large injury force, but both lower than parallel control, which indicates that it requires more time for the complete self-repair of the injured roots.

The self-repair of root is a complex process, which is not only related to the internal structure of the root system itself, but also influenced by various environmental factors such as soil water content and soil nutrients. In this research, we only selected roots from the same soil layer. As the physical and chemical properties of different soil layers are different, the roots in the different soil layers could have different self-repair characters. The characteristics of self-repaired roots in soils with varied depth, nutrients and moisture should be studied in the future.
